# Disease-modifying therapies are associated with improved olfactory function via NK cell- microbiota interactions in multiple sclerosis

**DOI:** 10.3389/fimmu.2026.1889149

**Published:** 2026-09-14

**Authors:** Zhuoma Danzhen, Zhang Yang, Lan Chu, Zidan Gao

**Affiliations:** 1 Department of Neurology, Affiliated Hospital of Guizhou Medical University, Guizhou Medical University, Guiyang, China; 2 Beijing Amcare Hospital, Jingshun Road, Chaoyang District, Beijing, China

**Keywords:** disease-modifying therapies (DMTs), lymphocyte subsets, multiple sclerosis, olfactory, university of pennsylvania smell identification test (UPSIT)

## Abstract

**Objective:**

To examine the associations of nasal microbiota with disease-modifying therapies (DMTs), lymphocyte subsets and olfactory function in multiple sclerosis (MS).

**Methods:**

Thirty patients with MS and 30 age- and sex-matched healthy controls (HCs) were enrolled. Patients were classified as DMT-treated (MS[DMT+]) or untreated (MS[DMT-]). Olfactory function was assessed using the University of Pennsylvania Smell Identification Test (UPSIT). Lymphocyte subsets were measured by flow cytometry, and nasal microbiota were analysed by 16S rRNA sequencing.

**Results:**

Olfactory function was lower in MS than in HCs, and better in MS(DMTs+) than in MS(DMTs−) (*p* =0.016, 95% CI 0.5614 to 4.941). NK cell counts were lower in MS(DMTs+) than in MS(DMTs−) (*p* =0.049, 95% CI -244.7 to -0.2109). And NK cell counts in MS(DMTs+) correlate with UPSIT (*r* =-0.527, *p* =0.029). Compared with HCs, MS(DMTs−) showed higher Shannon diversity (*p* =0.038), increased *Proteobacteria* (*p* =0.004), reduced *Moraxellaceae* (*p* =0.037) and *Enterobacteriaceae* (*p* =0.033), and enriched branched-chain amino acid biosynthesis (*p* =0.026). In MS(DMTs+), *Campylobacter, Anaerococcus, Peptoniphilus, Prevotella*, and *Finegoldia* were positively correlated with circulating NK cells (all *p*<0.05).

**Conclusion:**

DMTs may improve olfactory function in MS, possibly through modulation of circulating NK cell-associated immune status and nasal microbiota, in addition to controlling disease-related inflammation.

## Graphical Abstract

**Figure:**
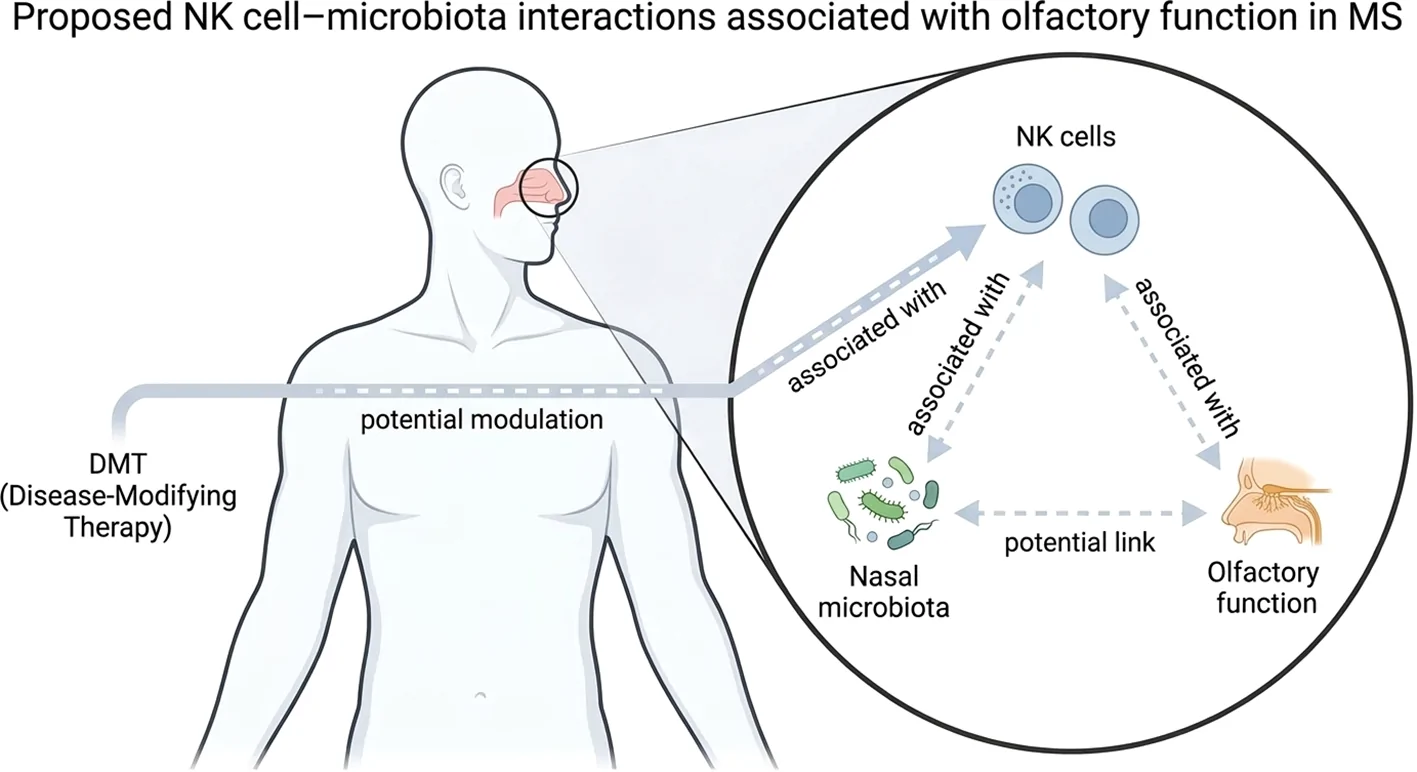
Proposed associations among olfactory function, DMT treatment, NK cells and nasal microbiota in multiple sclerosis. DMT treatment was associated with improved olfactory function and coordinated changes in peripheral immune status and the nasal microbiota. Arrows indicate associations rather than causality.

## 1 Introduction

Multiple sclerosis (MS) is a central nervous system disorder driven by autoimmune mechanisms, in which inflammatory activity results in demyelination and subsequent axonal impairment (1). It is one of the most common causes of non-traumatic disability among individuals aged 18–40 years, affecting an estimated 2.8 million people worldwide (2). Immune imbalance is a major contributor to MS pathogenesis, characterized by dysregulated expansion and infiltration of T and B lymphocytes (3). Patients during the remission phase are generally managed with disease-modifying therapies (DMTs). DMTs modulate immune cell activity, suppress lymphocyte-mediated responses, reduce relapse rates, and slow short-term disability progression (4). Some DMTs may also contribute to improved neurological function in MS (5). However, whether they ameliorate olfactory dysfunction remains unclear.

Olfactory function refers to the ability of an organism to detect and identify odors through the olfactory system (6). Patients with MS may experience olfactory dysfunction of varying severity, and recent evidence suggests that approximately 27.2% have olfactory impairment (7). However, the mechanisms underlying olfactory dysfunction in MS remain unclear. Abnormalities may occur at multiple sites along the olfactory pathway, and both olfactory bulb atrophy and neuroinflammation have both been reported in patients with MS (8). The nasal cavity is the first site of entry for odorant molecules, and the local nasal microbiota plays an important role in the proper development of the olfactory epithelium (9). Previous studies have reported a reduced abundance of *Corynebacterium* in patients with postviral olfactory dysfunction, suggesting that nasal microbiota dysbiosis may be associated with impaired olfactory function (10). The nasal microbiota is closely linked to olfactory function, and whether olfactory dysfunction in MS is associated with alterations in the nasal microbiota remains to be elucidated.

The nasal microbiota is located on the nasal epithelium, a key component of the body’s first line of immune defense (11). Host immune status and the nasal microbiotainfluence each other (12). The nasal microbiota contributes to maintaining the balance among Th1, Th2, and regulatory T cells (Tregs) (13). Colonization of the nasal cavity with *Lactobacillus murinus* and *Streptococcus pyogenes* may induce proinflammatory cytokine expression and modify the composition and abundance of circulating lymphocyte subsets (14). In MS, the gut microbiota has been implicated in shaping circulating lymphocyte profiles. DMT treatment has also been reported to be associated with alterations in the gut microbiota, suggesting potential bidirectional interactions between DMTs and gut microbial communities (15).

This study aimed to investigate the effects of DMTs on olfactory function in MS and to explore the underlying mechanisms, with a focus on circulating lymphocyte subsets, nasal microbiota, and their interactions.

## 2 Materials and methods

### 2.1 Participants

From February 2021 to March 2023, individuals diagnosed with multiple sclerosis (MS) were enrolled at the Affiliated Hospital of Guizhou Medical University. Diagnosis was based on the 2017 revised McDonald criteria, and disability was evaluated using the Expanded Disability Status Scale (EDSS) (16, 17). Eligibility criteria included an age range of 18–69 years and an EDSS score of 0–6.5. Baseline clinical characteristics, including sex, age, and and body mass index (BMI), were collected at enrollment.

Exclusion criteria were recent MS relapse (<30 days), comorbid neurological, psychiatric, nasal, or systemic disorders that could affect olfaction, head trauma or MRI-detected structural abnormalities, a history of upper respiratory tract infection within the preceding 3 weeks, prior radiotherapy or chemotherapy, substance dependence, menstruation or pregnancy, prolonged use (>1 month) of drugs that impair olfaction, an EDSS score exceeding 6.5, and an MMSE score below 24.

Ethical approval for this study was granted by the Clinical Ethics Committee of the Affiliated Hospital of Guizhou Medical University (approval Nos. 2021 Lun 495 and 2023 Lun 897). The study was performed in accordance with the principles outlined in the Declaration of Helsinki. All participants provided written informed consent prior to inclusion.

The dataset used in this study partly overlaps with a previously published cohort (18). However, the present analysis focused specifically on the effects of DMT exposure, which were not a primary focus of the previous publication.

### 2.2 Olfactory assessment methods

Olfactory performance was measured using the University of Pennsylvania Smell Identification Test (UPSIT), a validated method for quantitative assessment (19, 20). The validated simplified Chinese 40-item version with microencapsulated odorant strips was used (20). The UPSIT score was defined as the number of correctly identified odorants (0–40), and age- and sex-adjusted normative criteria were applied to classify the severity of olfactory dysfunction. All tests were administered by two trained investigators (20). Two trained researchers carried out the testing for all subjects and performed the statistical analyses.

### 2.3 Biospecimen collection

Nasal samples were collected using sterile swabs (LONGSEE, Guangzhou, China), which were inserted approximately 2–3 cm into the middle nasal passage and gently rotated for 5–10 s. After collection, each swab was transferred into 1 mL of sterile saline preservative and stored at −80°C for subsequent analysis.

### 2.4 Sequence analysis

Genomic DNA was extracted from human nasal swabs using the QIAamp DNA Mini Kit (QIAGEN). The V4 region of the 16S rRNA gene was amplified with primers 515F and 806R using Premix Taq (TaKaRa) under the following conditions: 94°C for 5 min; 30 cycles of 94°C 30 s, 52°C 30 s, 72°C 30 s; and 72°C for 10 min. Amplicons were purified and sequenced on the Illumina platform (PE250).

Raw reads were quality-trimmed using fastp (v0.14.1; sliding window: -W 4, -M 20) and primer-trimmed using cutadapt. Paired-end reads were merged with usearch v10 (-fastq_mergepairs; min overlap 16 bp, max mismatch 5 bp). OTUs were clustered at 97% identity using UPARSE, and chimeras, singletons, and sequences assigned to mitochondria or unclassified at the kingdom level were filtered out. Taxonomic classification was performed with SINTAX against the SILVA 138 database at a confidence threshold of 0.8; unclassified taxa at the genus level were labeled at the family level with an “unclassified” suffix.

Functional profiles were predicted using PICRUSt2, with annotations mapped to the KEGG Orthology (KO) and COG databases.

### 2.5 Percentages and absolute counts of lymphocyte subsets

Peripheral venous blood (2 mL) was collected from MS patients into heparinized tubes. For flow cytometry, 100 μL of whole blood was incubated for 30 min in the dark with fluorochrome-conjugated monoclonal antibodies against CD3, CD4, CD8, CD19, and CD16/CD56, including CD3-FITC, CD16/CD56-PE, CD19-APC, CD4-PerCP-Cy5.5, and CD8-BV421 (BD Biosciences, USA). Phosphate-buffered saline was obtained from Solarbio (Beijing, China), and all flow cytometry tubes and instruments were from BD Biosciences (USA).

### 2.6 Statistical analysis

Statistical analyses were performed using SPSS 24.0, GraphPad Prism 8.0.2, and R 3.5.1. Continuous variables were compared using unpaired two-tailed t-tests, and categorical variables were analyzed with the chi-square test or Fisher’s exact test, as appropriate. Pearson and Spearman correlation analyses were applied to assess relationships between clinical variables, lymphocyte subsets, and UPSIT scores. A two-sided p value < 0.05 was considered statistically significant.

For nasal microbiota analysis, α-diversity was evaluated using richness, Chao1, Shannon, and Simpson indices, and Beta diversity was assessed using Bray–Curtis dissimilarity and visualized by PCoA. Differences in community composition among groups were tested by PERMANOVA based on Bray–Curtis distance matrices using the Adonis *R*^2^. Taxonomic composition was summarized at the phylum and family levels, with taxa of relative abundance < 0.5% and unclassified taxa grouped as “Others”. Differential taxa were identified using LEfSe, and correlations between nasal microbiota and lymphocytes were visualized using heatmaps.

## 3 Results

### 3.1 Baseline characteristics

The baseline features of this cohort are outlined here and have been described in detail in our earlier report (18). The study included 30 individuals with MS and 30 healthy controls (HCs) matched for age and sex. No significant differences were found between MS and HC groups in sex distribution, age, BMI, or MMSE score (18). MS patients were stratified into untreated (MS(DMTs−), n = 13) and treated [MS(DMTs+), n = 17] groups. A significant difference in sex distribution was observed between subgroups (*p* = 0.01); however, other baseline demographic and clinical variables were comparable (**Table 1**). A subset of participants, comprising 14 healthy controls, 10 MS(DMTs+), and 3 MS(DMTs−) patients, underwent nasal microbiota analysis. The remaining participants were not included because of incomplete sample collection/insufficient sample volume/failure to meet predefined sample quality-control criteria/participant noncompliance (**Figure 1A**). In this study, the V3–V4 region of the 16S rRNA gene was sequenced in 27 nasal samples, generating a total of 3,349,902 raw paired-end reads. After quality filtering, sequence merging, and chimera removal, 3,136,801 high-quality clean tags were retained for downstream analyses. The mean number of clean tags per sample was 115,646 in the HC group, 116,518 in the MS (DMT+) group, and 117,526 in the MS (DMT−) group. The comparable sequencing depths across the three groups indicate that the sequencing data were sufficient for microbial composition and diversity analyses (**Supplementary Table 1**).

**Table 1:** Characteristics of MS patients with DMTs and without DMT .

Characteristics	MS (DMTs-) n=13	MS (DMTs+) n=17	Statistical values	*p*-V value
**Gender** ^b^ (Females/Males)	7/6	16/1	6.679^†^	0.01^*^
**Age** ^b^	31.00 (28.00-35.50)	34.00 (29.00-43.50)	-1.175^&^	0.240
**BMI** ^b^	22.27 (19.30-24.28)	20.96 (19.78-22.74)	-0.314^&^	0.754
**Age at onset** ^b^	28.00 (20.50-30.50)	30.00 (23.00-37.00)	-0.943^&^	0.346
**Disease duration** **^a^**	5.74 ± 1.3	4.96 ± 1.03	0.503^#^	0.619
**Number of relapses** **^a^**	2.92 ± 1.61	2.65 ± 1.62	0.465^#^	0.646
**EDSS Score** ^b^	2.00 (1.25-3.25)	2.00 (1.75-2.75)	-0.149^&^	0.881
Type of DMTs				
Teriflunomide		11		
Siponimod		4		
Dimethyl fumarate		1		
Rituximab Injection		1		
**UPSIT Score** **^a^**	26.31 ± 0.59	29.06 ± 0.82	-2.574^#^	0.016^*^

^a^Data are presented as mean ± standard error of the mean (SEM) or ^b^median (interquartile range, Q1–Q3), as appropriate.

*p < 0.05 indicates statistical significance.

**^#^**Comparisons between groups were performed using the independent-samples t-test for normally distributed variables.

^&^The Mann–Whitney U test (*Z* value) was used for non-normally distributed variables.

**^†^**The chi-square (*χ²*) test was applied for categorical variables.

MS, multiple sclerosis; BMI, body mass index; EDSS, Expanded Disability Status Scale; DMTs, Disease Modifying Therapies; UPSIT, University of Pennsylvania Smell Identification Test.

**Figure 1:**
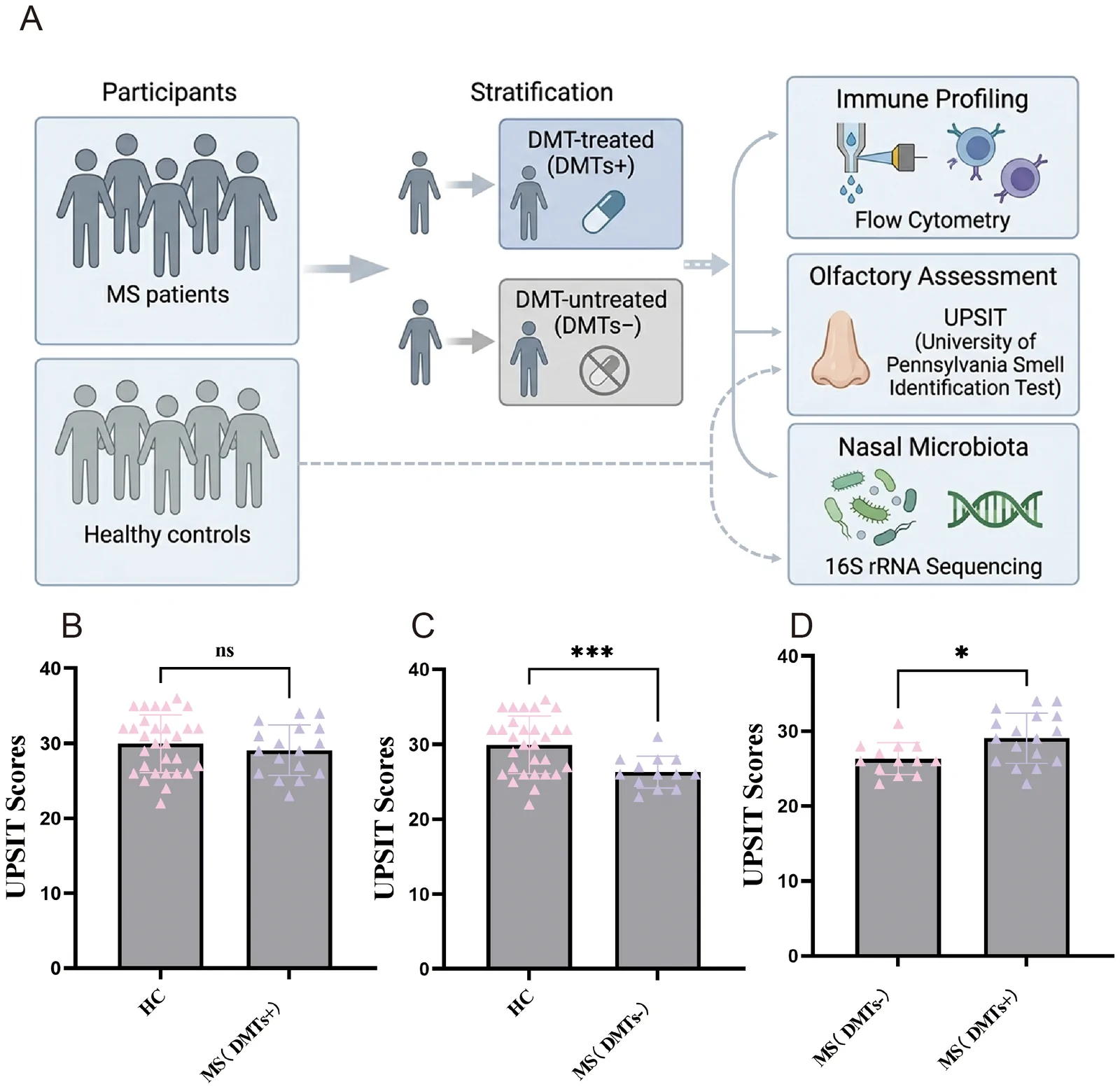
Study flowchart and UPSIT(University of Pennsylvania Smell Identification Test) analysis. **(A)** Study flowchart **(B)** Comparison of UPSIT scores between healthy controls (HC)(n=30) and patients with multiple sclerosis (MS)(n=30). **(C)** Comparison of UPSIT scores between HC(n=30) and MS (DMTs+)(n=17). **(D)** Comparison of UPSIT scores between HC and MS (DMTs−)(n=13). **(E)** Comparison of UPSIT scores between patients with MS (DMTs−)(n=13) and MS (DMTs+)(n=17). UPSIT scores are shown as bar charts and presented as mean ± standard deviation (SD). Statistical comparisons between groups were conducted using Student’s t-test. *p < 0.05, ***p < 0.001,ns= not significant. UPSIT, University of Pennsylvania Smell Identification Test; MS, multiple sclerosis; HC, healthy control; DMTs, disease-modifying therapies.

### 3.2 Olfactory function

Olfactory function was assessed using UPSIT. The mean UPSIT scores in individuals with MS were significantly reduced compared to those in HCs [27.87 ± 3.10 vs. 29.93 ± 3.84, *p* = 0.027, 95% CI: (−3.887 to −0.247)] (18). There was no significant difference between MS (DMTs+) and HCs (*p* = 0.437; **Figure 1B**), whereas MS(DMTs−) showed significantly lower UPSIT scores than HCs [29.93 ± 3.84 vs. 26.31 ± 0.59, *p* < 0.001,95% CI: (−5.484 to −1.767), **Figure 1C**]. Among patients with MS, those in the MS (DMTs−) group had significantly lower UPSIT scores compared with the MS (DMTs+) group [26.31 ± 0.59 vs. 29.06 ± 0.82, *p* = 0.016, 95% CI: (0.5614 to 4.941) **Table 1**; **Figure 1C**].

### 3.3 Lymphocyte subsets

Lymphocyte subset counts and proportions were compared between MS(DMTs−) and MS(DMTs+). Absolute counts of total T cells [*p* = 0.001, 95% CI: (307.009 to 1169.162)], CD8^+^ T cells [*p* = 0.002, 95% CI: (96.554 to 395.743)], CD4^+^ T cells [*p* = 0.001, 95% CI: (214.720 to 807.496)], and B cells (*p* < 0.001) were significantly lower in MS(DMTs+) than MS(DMTs−) (**Table 2**; **Figures 2A–C, E**). Absolute NK-cell counts were also lower in MS(DMTs+) [*p* = 0.049, 95% CI: (-244.7 to -0.2109) (**Table 2**; **Figure 2F**)]. There was no difference in the CD4/CD8 ratio among the groups (*p* = 0.739; **Table 2**; **Figure 2D**). In contrast, the combined proportion of T+B+NK% was significantly lower in MS(DMTs+) than in MS(DMTs-) (*p* = 0.018; **Table 2**). NK-cell proportions tended to be higher in MS(DMTs+), though not significantly (*p* = 0.098; **Figures 2G, H**). MS (DMTs+) patients exhibited lower proportions of total T cells, CD8^+^ T cells, CD4^+^ T cells, and B cells, but these differences were not significant (**Figures 2G, H**).

**Table 2:** Lymphocyte subgroup test results of MS patients.

Characteristics	MS (DMTs-) n=13	MS (DMTs+) n=17	Statistical values	*p*-V value
T Cells ^a^	1661.62 ± 173.85	923.53 ± 127.48	3.507	0.001^**^
CD8^+^T Cells ^a^	596.39 ± 52.43	350.23 ± 49.68	3.371	0.002^**^
CD4^+^ T Cells^a^	976.46 ± 138.57	465.35 ± 69.74	4.940	0.001^**^
NK Cells ^a^	332.39 ± 53.35	209.94 ± 21.90	2.123^†^	0.049^*^
B Cells ^b^	241.00 (187.00-380.00)	138.00 (45.00- 188.00)	-3.327^†^	<0.001^***^
CD4/8 ^a^	1.72 ± 0.23	1.60 ± 0.26	0.336	0.739
T+B+NK % ^b^	99.84(99.75-99.93)	99.73 (99.50-99.81)	-2.349^†^	0.018^*^
T Cells % ^a^	72.89 ± 1.88	68.55 ± 3.25	1.064	0.296
CD8^+^TCells %^a^	28.15 ± 2.99	26.99 ± 2.25	0.314	0.756
CD4^+^ T Cells %^a^	41.31 ± 2.57	34.49 ± 3.24	1.573	0.127
NK Cells % ^a^	14.34 ± 1.88	20.16 ± 2.73	-1.712	0.091
B Cells % ^a^	12.64 ± 1.29	10.12 ± 1.34	1.318	0.198

^a^Data are presented as mean ± standard error of the mean (SEM) or ^b^median (interquartile range, Q1–Q3), as appropriate.

*p < 0.05, **p < 0.01, ***p < 0.001 indicate statistical significance.

^†^The Mann–Whitney *U* test (*Z* value) was used for non-normally distributed variables.

All other continuous variables were analyzed using the independent-samples *t*-test.

MS, multiple sclerosis; DMTs, disease-modifying therapies.

**Figure 2:**
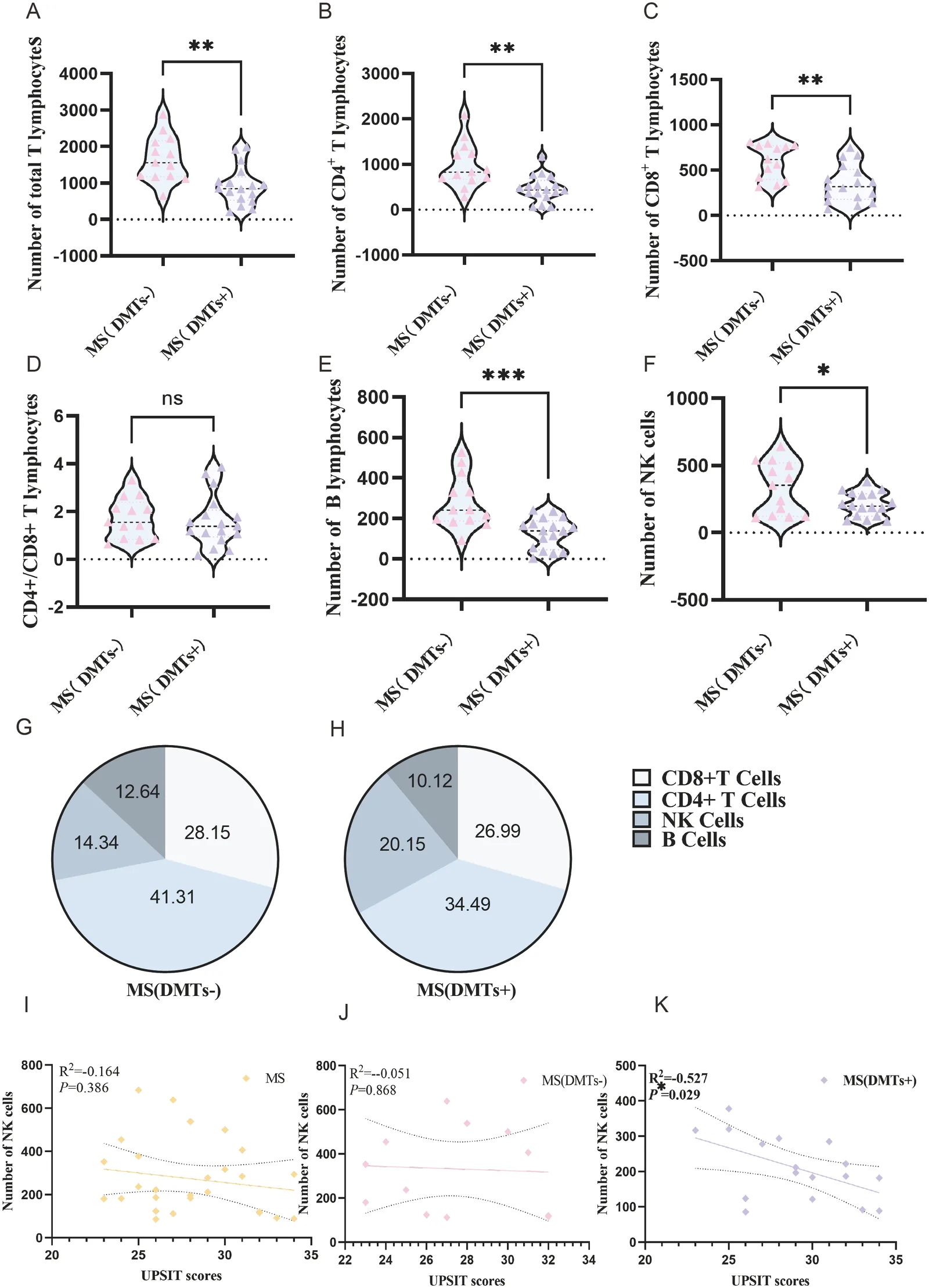
Lymphocyte subset analysis. **(A–F)** Violin plots showing differences in peripheral lymphocyte subsets between MS(DMTs−)(n=13) and MS(DMTs+)(n=17) patients, including total T cells **(A)**, CD4+ T cells **(B)**, CD8+ T cells **(C)**, CD4+/CD8+ ratio **(D)**, B cells **(E)**, and NK cells **(F)**; individual values are shown, data are presented as mean ± SD, and linear regression lines with 95% CI (dotted) are indicated. **(G, H)** Pie charts showing the relative proportions of major lymphocyte subsets in the MS(DMTs−) group **(G)** and the MS(DMTs+) group **(H)**, including CD8+ T cells, CD4+ T cells, NK cells, and B cells.**(I–K)** Correlation analyses between UPSIT scores and NK cell counts in all patients with MS **(I)**, in the MS(DMTs−) group **(J)**, and in the MS(DMTs+) group **(K)**. Statistical significance is indicated as follows: *p < 0.05, **p < 0.01, ***p < 0.001. ns, not significant. UPSIT, University of Pennsylvania Smell Identification Test; DMT, disease-modifying therapy; NK, natural killer.

### 3.4 UPSIT and lymphocyte subsets

Correlation analysis was used to examine the relationship between olfactory performance and lymphocyte subsets in MS. In the overall MS group, UPSIT scores were not significantly correlated with NK-cell absolute counts (*r* = -0.164, *p* = 0.386; **Figure 2I**) or in the MS(DMTs−) subgroup (*r* = -0.051, *p* = 0.868; **Figure 2J**). By comparison, the MS (DMTs+) subgroup showed a significant inverse correlation (*r* = −0.527, *p* = 0.029; **Figure 2K**). There were no significant correlations between UPSIT scores and the absolute numbers of total T cells, CD8^+^ T cells, CD4^+^ T cells, or B cells (**Supplementary Figure 1**).

### 3.5 Nasal microbiota

#### 3.5.1 α- and β-diversity

For α-diversity, Chao1 richness (*p* = 0.753), Shannon index(*p* = 0.104), and Simpson index(*p* = 0.145) were not significant in HCs and overall MS^17^.Chao1 richness did not differ significantly among HCs vs. MS(DMTs+); HCs vs. MS(DMTs−); and MS(DMTs+) vs. MS(DMTs−) (all *p* >0.05) (**Figure 3A**). Conversely, healthy controls (HCs) showed a significantly higher Shannon index compared with the MS (DMTs−) group (*p* = 0.038), whereas not significant in the HCs vs. MS(DMTs+), and MS(DMTs+) vs. MS(DMTs−),(all *p* >0.05); (**Figure 3B**). The Simpson index was significantly lower in HCs than in MS(DMTs−) (*p* = 0.023), but not in the other comparisons (HCs vs. MS(DMTs+), MS(DMTs+) vs. MS(DMTs−), all *p* >0.05); (**Figure 3C**).

**Figure 3:**
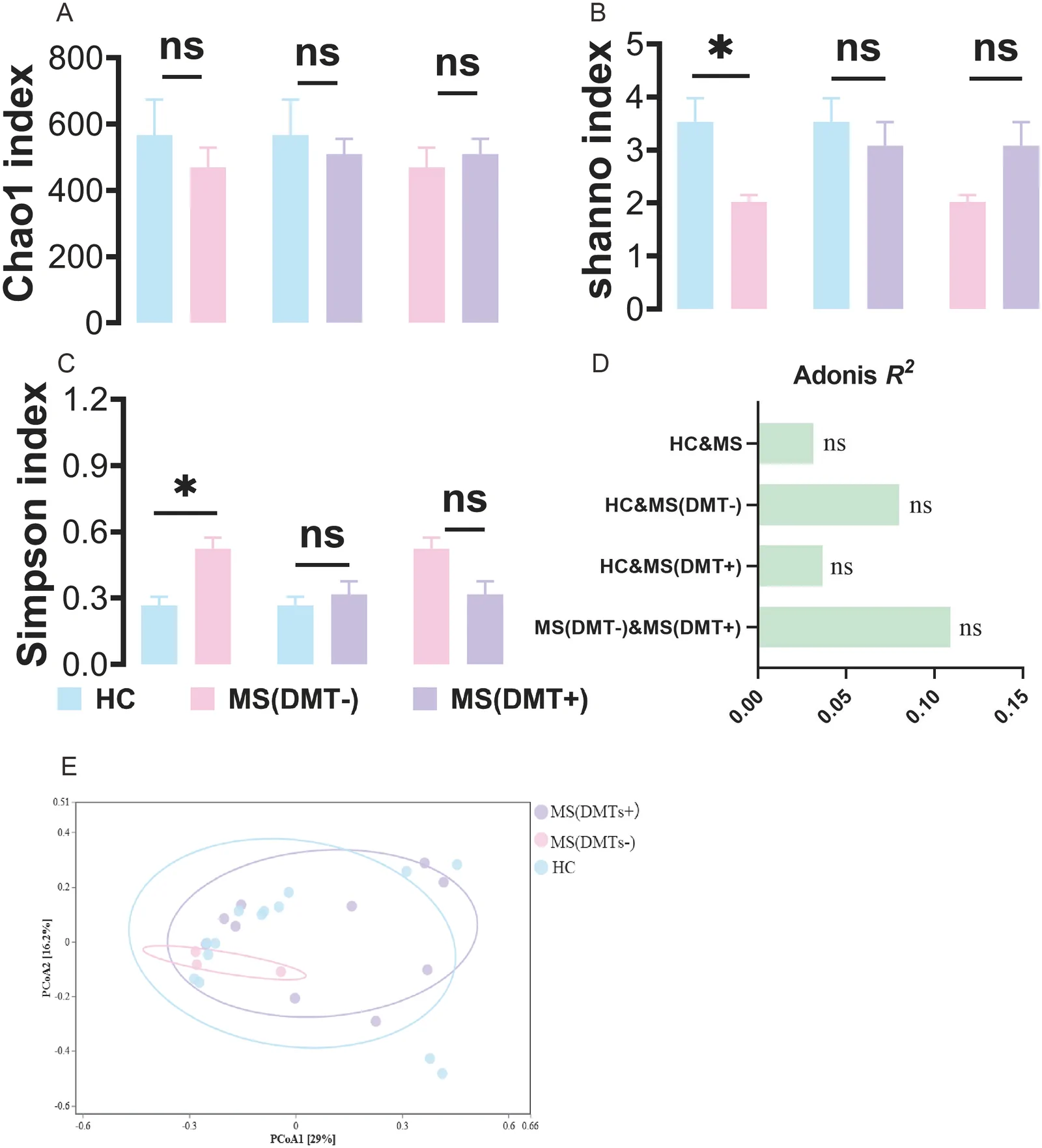
Alpha and beta diversity of nasal bacterial microbiomes. **(A–C)** Comparison of alpha diversity among the healthy control (HC), (MS),MS(DMTs−), and MS(DMTs+) groups, as assessed by the Chao1 index **(A)**, Shannon index **(B)**, and Simpson index **(C)**. **(D)** Pairwise permutational multivariate analysis of variance (PERMANOVA) based on beta diversity, with Adonis *R*^2^ values shown for comparisons between HC and MS, HC and MS(DMTs−), HC and MS(DMTs+), and MS(DMTs−) and MS(DMTs+).**(E)** Principal coordinate analysis (PCoA) was used to visualize HC, MS(DMTs−), and MS(DMTs+) groups microbiome with Bary–Curtis community distance and the distance between samples reflected the similarity of microbial community structure. Ellipses indicate the distribution of samples within each group. Pairwise comparisons were performed using one-way ANOVA, or Welch’s ANOVA when the assumption of homogeneity of variances was not met. Statistical significance is indicated as follows: *p < 0.05; ns, not significant. HC, healthy controls; DMT, disease-modifying therapy; PCoA, principal coordinate analysis.

For β-diversity, PERMANOVA confirmed no significant difference in HCs vs. MS (*R²* = 0.0316, *p* = 0.658; **Figure 3D**). After stratification by DMT exposure, no significant differences were detected among all the following groups: HCs vs. MS(DMTs+), HCs vs. MS(DMTs−), MS(DMTs+) vs. MS(DMTs−) (all *p* > 0.05) (**Figure 3D**). Bray-Curtis-based PCoA showed no significant separation between HCs, MS(DMTs+)and MS(DMTs−) (**Figure 3E**) (all *p* >0.05).

#### 3.5.2 Differential abundance

At the phylum level, we presented the top 20 abundant taxa, comparing HC vs. MS (**Figures 4A** left), MS(DMT+) vs. MS(DMT−) (**Figures 4A** middle), and HC vs. MS(DMT−) (**Figures 4A** right). Differential analysis revealed *Proteobacteria* was more abundant in MS(DMTs−) than HCs (*p* = 0.004, 95% CI [-0.1708 to -0.040], two-sided unpaired Welch’s t test; **Figure 4B**).

**Figure 4:**
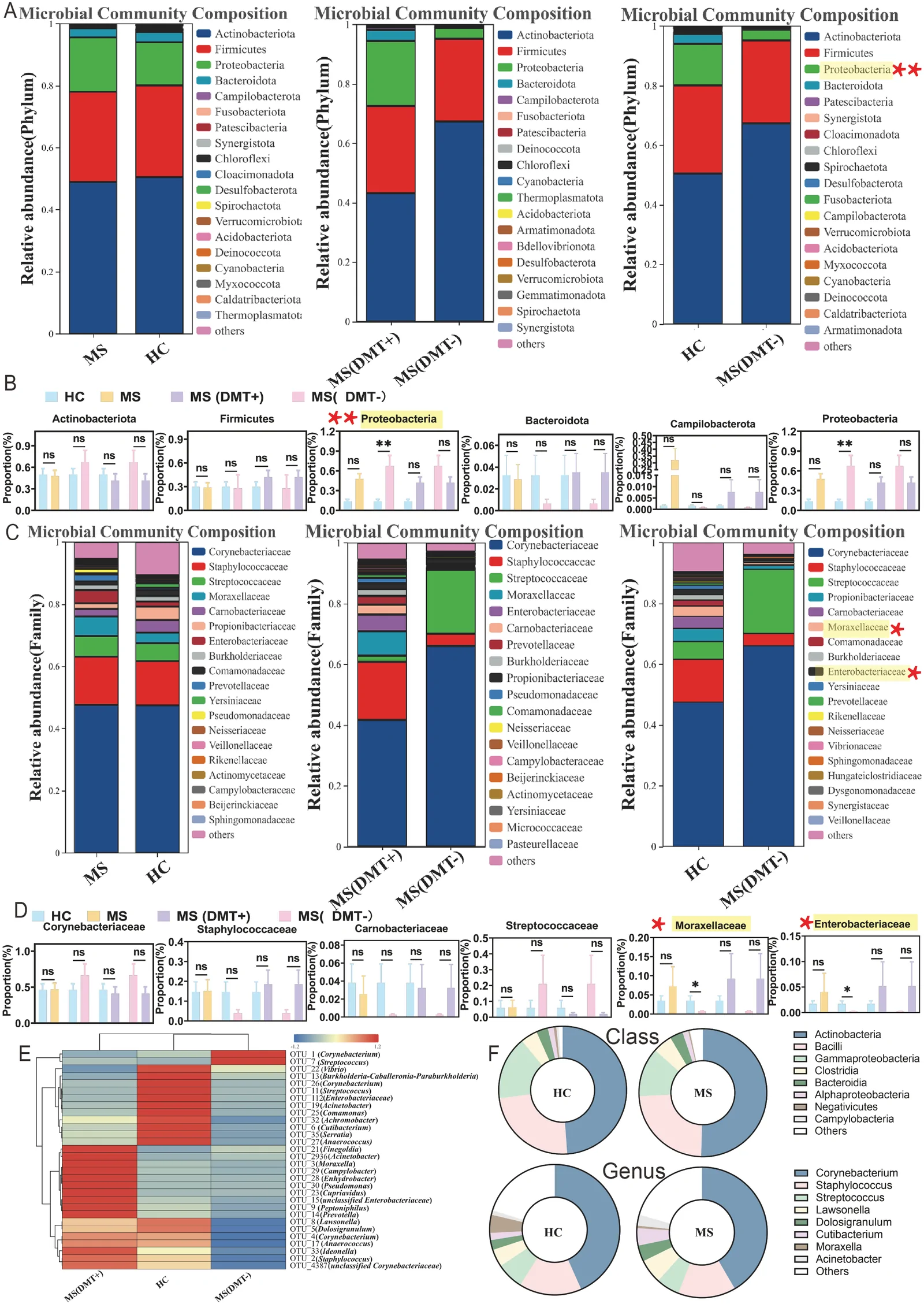
Alterations in nasal microbial community and differential taxa across groups. **(A)** Relative abundance of Top20 microbial communities at the Phylum level across groups [MS, HC, MS(DMT+), and MS(DMT−)]. **(B)** Exploratory differential abundance analysis of major microbial phyla among groups. **(C)** Relative abundance of Top20 microbial communities at the Family level across groups. **(D)** Exploratory differential abundance analysis of representative microbial families across the groups. **(E)** Heatmap with hierarchical clustering based on relative abundance of microbial taxa, illustrating compositional differences and similarities among samples. **(F)** Pie charts showing the relative distribution of dominant taxa at the Class and Genus levels in HC and MS groups. For differential abundance analysis **(B, D)**, statistical comparisons were performed using the Kruskal–Wallis test followed by Dunn’s *post hoc* multiple comparisons test. *P*-values were adjusted for multiple testing using the false discovery rate (FDR) method (Benjamini–Hochberg correction). Data are presented as mean ± standard error of the mean (SEM). Statistical significance is indicated as *p < 0.05, **p < 0.01, while “ns” indicates no significant difference.

At the family level, we presented the top 20 abundant taxa, comparing HC vs. MS (**Figures 4C** left), MS(DMT+) vs. MS(DMT−) (**Figures 4C** middle), and HC vs. MS(DMT−) (**Figures 4C** right). Statistical analysis using the Wilcoxon rank-sum test showed *Moraxellaceae* (*p* = 0.037, 95% CI [-0.055 to -0.002]) and *Enterobacteriaceae* (*p* = 0.033, 95% CI [-0.028 to -0.001]) were significantly less abundant in MS(DMTs−) than HCs (**Figure 4D**). Comparisons among the three groups at the phylum and family levels are presented in **Supplementary Figure 2**. OTU profiles in HCs and MS(DMT−) were similar overall and clustered together (**Figure 4E**), while MS(DMT+) formed a separate branch. Several OTUs (e.g., *Corynebacterium, unclassified Enterobacteriaceae*) were enriched in untreated MS patients but decreased after DMT treatment. With comparable class- and genus-level community structures (**Figure 4F**).

#### 3.5.3 Differential taxa and predicted functions

LEfSe analysis identified differential taxa across groups. In the HC vs. MS(DMTs+) comparison, 11 taxa were significantly different (LDA score > 2.0, p < 0.05), with 9 (*Acetoanaerobium* sp., *Bacillus* sp. TX3, *Flavihumibacter cheonanensis*, *Leptotrichia* sp. PG10, *Marinococcus* sp. GSP32, *Nocardioides* sp. S2_84, *Paenibacillus* sp. YJ01, *Selenomonas lacticifex*, *Sphingobacterium thalpophilum*)enriched in MS(DMTs+) and 2(*Azospirillum* sp. CRRI_31, *Sphingomonas* sp. 2D232011) in HCs (all *p* < 0.05) (**Figure 5A**). In HC vs. MS(DMTs−), 16(*Bacillus* sp. TX3, *Chromobacterium alkanivorans*, *Chryseobacterium* sp. LW_NC3, *Jaaginema geminatum* SAG 1459-8, Lachnospiraceae bacterium 610, *Lagierella massiliensis*, *Marinococcus* sp. GSP32, *Microlunatus lacustris*, *Ndongobacter massiliensis*, *Nevskia ramosa*, *Pleurocapsa* sp. HA4230, *Polyangium* sp. CC_YHH645, *Sphingobacterium thalpophilum*, uncultured *Actinomyces* sp., uncultured *Hymenobacter* group bacterium, uncultured actinobacterium) different taxa were detected, most enriched in MS(DMTs−), indicating more pronounced microbial changes in untreated patients (all *p* < 0.05) (**Figure 5A**). The MS(DMTs+) vs. MS(DMTs−) comparison identified 4 different taxa(*Bacteroidetes bacterium* Oral Taxon 274, *Chryseobacterium* sp. LW_NC3, *Lagierella massiliensis*, *Nesterenkonia* sp. MCCC 1A10687), all enriched in MS(DMTs−), suggesting partial modulation of microbial composition by DMT treatment (all *p* < 0.05) (**Figure 5A**).

**Figure 5:**
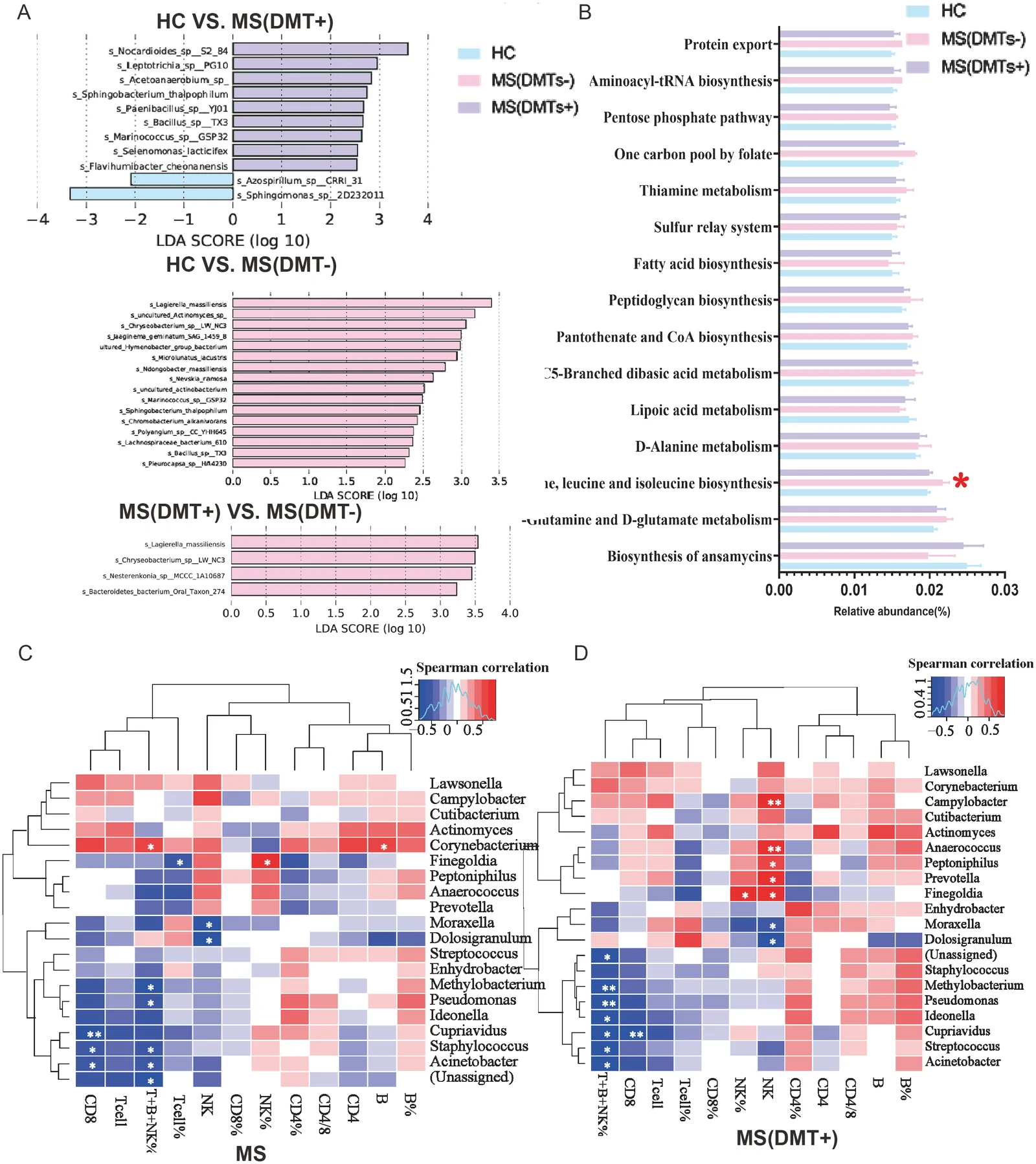
Functional profiling and differential pathway analysis of gut microbiota among groups. **(A)** LEfSe analysis using Kruskal–Wallis and Wilcoxon rank-sum tests with LDA identified significantly enriched taxa between HC and MS(DMT−), HC and MS(DMT+), and MS(DMT−) and MS(DMT+); only taxa with an LDA score > 2.0 are shown. **(B)** Relative abundance of predicted microbial functional pathways among groups based on KEGG annotation; statistical significance was assessed using the Kruskal–Wallis test followed by Dunn’s multiple comparisons test, with differential pathways between groups highlighted. **(C, D)** Spearman correlation heatmaps showing associations between nasal microbial genera and peripheral immune cell subsets in MS patients [**(C)** untreated; **(D)** DMT-treated], with colors indicating correlation strength (red, positive; blue, negative; white, near zero); statistical significance was assessed using the Kruskal–Wallis test followed by Dunn’s multiple comparisons test. Statistical significance is indicated as *p < 0.05, **p < 0.01; “ns” indicates no significant difference.

Functional prediction based on KEGG showed that, compared with HCs the MS(DMTs−) had a significantly higher relative abundance of the valine, leucine and isoleucine biosynthesis pathway (*p* = 0.026; **Figure 5B**). The other pathways showed no statistically significant differences. Functional prediction based on COG databases (**Supplementary Figure 3**).

### 3.6 Associations between lymphocyte subsets and the nasal microbiota

Spearman correlation heatmaps were used to assess relationships between nasal microbial genera and peripheral immune cell subsets in MS and MS(DMTs+) patients (**Figures 5C, D**). Color intensity reflects the strength of correlations (red indicating positive and blue indicating negative), while statistically significant associations are marked (*p < 0.05; **p < 0.01).*Campylobacter, Anaerococcus, Peptoniphilus, Prevotella*, and *Finegoldia* exhibited positive correlations with NK cell count(all *p* <0.05). These taxa were uniquely associated with NK cells in MS (DMT+) compared to the MS group analyzed earlier. No significant associations were observed in the MS(DMTs−) subgroup, which may reflect the limited sample size of the untreated group (**Supplementary Figure 4**).

## 4 Discussion

This study provides evidence, for the first time, that DMTs may be associated with improvements in olfactory function in patients with MS, accompanied by coordinated changes in immune status and the nasal microbiota. These findings extend the recognised immunomodulatory effects of DMTs and suggest a microbiota-related regulatory pathway involved in olfactory recovery.

MS patients receiving DMT scored higher on the UPSIT compared to untreated patients, suggesting that olfactory function could serve as a sensitive indicator for monitoring treatment effects. This also helps explain the wide variation in the prevalence of olfactory dysfunction observed in previous studies, with reported rates ranging from 27.2% to 93% (7, 21). While methodological and clinical differences contribute to this, our findings suggest DMT treatment could be an overlooked confounding factor. Future assessments of olfactory dysfunction in MS should consider DMT use, treatment type, and duration.

Previous studies suggest that changes in olfactory function in MS may reflect inflammatory status and serve as potential biomarkers of treatment response (22). The olfactory improvement associated with DMTs may involve multiple mechanisms. DMTs could reduce excessive immune activation and pro-inflammatory mediators (23), thereby alleviating neuroinflammatory damage in the olfactory pathways, although this mechanism requires further validation. DMTs may indirectly shape the nasal microbiome through immune modulation. Consistently, untreated patients showed significant changes in microbial diversity and composition compared with healthy controls. Compared with healthy controls, untreated MS patients exhibited a significantly decreased Shannon index and an increased Simpson index. The Shannon index comprehensively evaluates alpha diversity by integrating species richness and community evenness; its reduction in untreated MS patients indicates a diminished pool of microbial species and less evenly distributed taxa within the nasal cavity. In contrast, the Simpson index is more sensitive to dominant microbial populations, and its elevation reflects the over-proliferation of a small number of dominant strains and imbalanced community distribution. These concordant shifts in the two diversity indices jointly demonstrate reduced richness and skewed dominance of the nasal microbiota in untreated MS patients. The elevated levels of *Proteobacteria* may indicate immune activation and ongoing inflammation. *Proteobacteria* has been shown to significantly increase in nasal inflammatory diseases such as chronic rhinosinusitis (24). The reduction of *Moraxellaceae* and *Enterobacteriaceae* may indicate depletion of commensal bacteria that sustain physiological nasal mucosal homeostasis under healthy conditions. *Moraxellaceae* constitute core commensal taxa of the upper respiratory tract, and their abundance exhibits bidirectional correlations with local airway inflammation and mucosal immune status: physiological colonization maintains immune balance, whereas excessive proliferation drives inflammatory lesions. A recent large-scale nasal microbiome genome-wide association study demonstrated that host genetic variation and multiple host-related and lifestyle factors contribute to variation in nasal microbial community composition (25). In addition, *Moraxella catarrhalis* has been shown to induce nasal airway epithelial damage and increase the expression of the pro-inflammatory cytokines IL-8 and IL-33, supporting a potential role for this species in contributing to mucosal inflammatory responses (26). Alterations in *Enterobacteriaceae* are frequently reported as a hallmark of nasal microbial dysbiosis (27). These two bacterial families are tightly associated with the immune microenvironment of nasal mucosa. In our study, these differences were no longer observed after DMT treatment, suggesting that DMT may reshape the immune microenvironment by restoring nasal microbial homeostasis. Immune dysregulation may contribute to dysbiosis of the upper respiratory microbiota, whereas a well-regulated mucosal immune environment helps maintain microbial homeostasis (28). Conversely, a balanced microbial community may help preserve olfactory epithelial homeostasis and support its repair and regeneration through microbe–host interactions, including bioactive metabolites and regulation of local inflammation (29). Functional prediction analyses revealed metabolic adaptation in untreated MS patients, as evidenced by the enrichment of branched-chain amino acid (BCAA) biosynthesis pathways. As BCAAs can influence both microbial community structure and Th17 cell differentiation via mTOR signaling, this finding may indicate a shift toward a pro-inflammatory metabolic profile (30). Consistent with the above metabolic and microbial findings, our prior research has identified two key features in patients with multiple sclerosis: significant shifts in nasal commensal composition, as well as a broken correlation between olfactory function (quantified by UPSIT scores) and superior turbinate morphological volume. Together, these earlier observations indicate that disrupted crosstalk between nasal mucosa and resident microbiota is maybe one of critical driver of MS-related olfactory loss (18). Building on this evidence, we hypothesize that DMTs exert reciprocal regulatory impacts on both nasal mucosal immune homeostasis and upper respiratory microbial communities. DMT treatment was associated with reductions in T cells, CD4^+^ T cells, CD8^+^ T cells, B cells and NK cells, indicating broad immunomodulatory effects. However, the included DMTs may differ in their effects on NK cells. Most DMT-treated participants received teriflunomide, which primarily inhibits activated T- and B-cell proliferation and is generally considered to have a relatively limited direct effect on NK cells. Siponimod may reduce circulating NK-cell counts, whereas dimethyl fumarate and rituximab are also thought to exert comparatively limited direct effects on NK cells. Therefore, the observed NK-cell alterations may not be fully attributable to DMT exposure alone. Associations between multiple nasal taxa and T cells, CD8^+^ T cells, B cells and NK cells, observed in both the overall MS cohort and the DMT-treated subgroup, further suggest an interaction between peripheral immune status and the nasal microenvironment. Notably, in the DMT-treated group, NK cell counts were inversely associated with UPSIT scores, while *Campylobacter, Anaerococcus, Peptoniphilus, Prevotella* and *Finegoldia* showed positive associations with NK cells, supporting a potential role for NK cell-related immune status in linking specific nasal taxa to olfactory function. Previous studies offer partial support for these observations, with *Campylobacter* linked to poorer olfactory thresholds (31) and *Anaerococcus* enriched in anosmic individuals (32). In contrast, evidence for *Peptoniphilus* remains limited, and *Finegoldia* has not been directly associated with olfactory scores, although its pro-inflammatory properties suggest possible indirect relevance. Although direct evidence that NK cells mediate olfactory epithelial injury is lacking, activated NK cells may induce mucosal epithelial damage through granzyme B release and perforin-dependent cytotoxicity (33). These findings suggest that NK cells may represent a potential immunological link between DMT treatment, nasal microbial changes and olfactory dysfunction, although the mechanisms involved and the roles of distinct NK cell subsets remain to be clarified. Nevertheless, because the individual DMT subgroups were small and unevenly distributed, particularly for siponimod, dimethyl fumarate and rituximab, treatment-specific effects on NK cells could not be evaluated, and residual confounding by DMT exposure cannot be excluded.

Several limitations of this study should be acknowledged. The sample sizes of several subgroups were small. The untreated MS group included only three patients; therefore, findings related to this subgroup should be interpreted cautiously and regarded as exploratory. The dimethyl fumarate and rituximab subgroups each included only one patient, precluding reliable drug-specific conclusions and highlighting the need for validation in larger, adequately powered cohorts. Although available immunological evidence suggests that the DMTs included in this study have limited direct effects on NK cells, supporting our interpretation that nasal immune alterations may be driven primarily by microbial remodelling rather than direct drug-induced immunosuppression, this hypothesis requires confirmation in larger studies with more balanced representation across DMT subgroups.

In summary, DMT treatment may be associated with improved olfactory function in MS, accompanied by changes in peripheral immunity and the nasal microbiota. The inverse correlation between NK cell counts and UPSIT scores, along with their associations with specific nasal taxa, suggests a potential link among immune modulation, microbial balance, and olfactory function. Further studies are needed to clarify the underlying mechanisms.

## Data Availability

The raw 16S rRNA gene amplicon sequencing data generated in this study have been deposited in the National Microbiology Data Center (NMDC) under accession number, NMDC10020881. (https://nmdc.cn/resource/genomics/project/detail/NMDC10020881).
