# Supplementary material for: Disease-modifying therapies are associated with improved olfactory function via NK cell- microbiota interactions in multiple sclerosis
Source: Front Immunol. 2026 Sep 14;17:1889149. doi: 10.3389/fimmu.2026.1889149 (PMC13617127; doi:10.3389/fimmu.2026.1889149)
Supplement: Supplementary file 1 [file SupplementaryFile1.doc]

**Supplementary Figure1**

a.b.c. MS, MS (DMTs+ ) , MS (DMTs-) UPSIT correlation analysis MS with absolute T cell counts

d.e.f. MS, MS (DMTs+ ) , MS (DMTs-) UPSIT correlation analysis MS with absolute CD8^+^T cell counts

g.h.i. MS, MS (DMTs+ ) , MS (DMTs-) UPSIT correlation analysis MS with absolute CD4^+^T cell counts

j.k.l. MS, MS (DMTs+ ) , MS (DMTs-) UPSIT correlation analysis MS with absolute B cell counts

**Supplementary** Table 1. Summary of 16S rRNA sequencing reads across study groups

| Sequencing | SampleID | Raw_total_reads | Clean_total_reads | Clean_total_tags | Q20(%) | Q30(%) | GC(%) |
| --- | --- | --- | --- | --- | --- | --- | --- |
| paired-end | HC1 | 126445 | 126185 | 120368 | 98.7 | 95.3 | 53.3 |
| paired-end | HC2 | 107595 | 107370 | 102986 | 98.8 | 95.7 | 54.8 |
| paired-end | HC3 | 117054 | 116810 | 111277 | 98.7 | 95.2 | 54 |
| paired-end | HC4 | 131522 | 131230 | 125286 | 98.8 | 95.5 | 54.3 |
| paired-end | HC5 | 120293 | 119913 | 113152 | 98.8 | 95.5 | 54.9 |
| paired-end | HC6 | 125822 | 125495 | 117278 | 98.7 | 95.4 | 53.9 |
| paired-end | HC7 | 131769 | 131498 | 124581 | 98.7 | 95.3 | 54.5 |
| paired-end | HC8 | 123535 | 121801 | 109455 | 98.3 | 94.1 | 52.6 |
| paired-end | HC9 | 120457 | 118748 | 105154 | 97.8 | 93.1 | 52.6 |
| paired-end | HC10 | 131588 | 131272 | 122620 | 98.4 | 94.5 | 52.7 |
| paired-end | HC11 | 128953 | 128650 | 122284 | 98.7 | 95.2 | 54.3 |
| paired-end | HC12 | 123824 | 123537 | 116803 | 98.6 | 95.1 | 54 |
| paired-end | HC13 | 126819 | 126529 | 118228 | 98.8 | 95.6 | 54 |
| paired-end | HC14 | 121024 | 120587 | 109571 | 98.3 | 94.3 | 50.5 |
| paired-end | MS(DMT+)1 | 128175 | 127922 | 119701 | 98.8 | 95.6 | 54.7 |
| paired-end | MS(DMT+)2 | 120499 | 120145 | 112409 | 98.5 | 94.8 | 51.7 |
| paired-end | MS(DMT+)3 | 125171 | 124888 | 118947 | 98.8 | 95.5 | 54.7 |
| paired-end | MS(DMT+)4 | 120208 | 119920 | 113751 | 98.7 | 95.3 | 53.7 |
| paired-end | MS(DMT+)5 | 125881 | 125576 | 119539 | 98.6 | 95.1 | 54.4 |
| paired-end | MS(DMT+)6 | 124609 | 124216 | 116748 | 98.5 | 94.8 | 52.3 |
| paired-end | MS(DMT+)7 | 115315 | 115079 | 109865 | 98.8 | 95.5 | 53.6 |
| paired-end | MS(DMT+)8 | 127202 | 126907 | 121449 | 98.8 | 95.5 | 53.8 |
| paired-end | MS(DMT+)9 | 122933 | 122672 | 117475 | 98.7 | 95.4 | 53.6 |
| paired-end | MS(DMT+)10 | 128753 | 128501 | 115296 | 98.6 | 95.1 | 50.4 |
| paired-end | MS(DMT−)1 | 126283 | 125997 | 120011 | 98.8 | 95.5 | 55.2 |
| paired-end | MS(DMT−)2 | 121807 | 121548 | 115528 | 98.5 | 94.9 | 53 |
| paired-end | MS(DMT−)3 | 126366 | 126047 | 117039 | 98.7 | 95.3 | 53.7 |

**Supplementary Figure 2.**

A.Relative abundance of Top20 microbial communities at the Phylum level across groups (MS, HC, MS(DMT+), and MS(DMT−)). B.Relative abundance of Top20 microbial communities at the Family level across groups (MS, HC, MS(DMT+), and MS(DMT−)).

**Supplementary Figure 3.**

GC-O pathway analysis showing the relative abundance of functional categories in nasal microbiota among healthy controls (HC), MS patients treated with DMT (MS(DMT+)), and untreated MS patients (MS(DMT-)). Each bar represents the proportion of sequences assigned to a specific functional category. Pathways are labeled according to the KEGG orthology (e.g., [J] Translation, ribosomal structure and biogenesis; [E] Amino acid transport and metabolism; [G] Carbohydrate transport and metabolism; [R] General function prediction only; etc.). Overall, the relative abundance of functional categories is broadly similar across groups, with minor differences observed in pathways such as [J] Translation and ribosomal structure, and [G] Carbohydrate transport and metabolism

**Supplementary Figure 4.**

Correlation heatmaps were used to analyze the correlation of MS (DMTs-) communities with the percentage of lymphocyte subpopulations versus absolute counts.
